# Digital application competence among adults with disabilities in South Korea: associations with latent profiles of digital attitudes and digital self-efficacy

**DOI:** 10.3389/fpsyg.2026.1830991

**Published:** 2026-06-10

**Authors:** Junyue Yue, Kyoung Ok Kim, Yeong Hun Yeo

**Affiliations:** 1 Jeonbuk National University, Jeonju, Republic of Korea; 2 Jeonju Seowon Senior Club, Social Welfare Foundation Geumsansa Welfare Center, Jeonju, Republic of Korea

**Keywords:** adults with disabilities, digital application competence, digital attitudes, digital self-efficacy, latent profile analysis

## Abstract

**Introduction:**

As public services in South Korea rapidly digitalize, adults with disabilities remain at heightened risk of digital exclusion beyond access barriers. This study aims to identify joint profiles of digital attitudes and digital self-efficacy among adults with disabilities and examine their associations with digital application competence. We further test whether these associations differ by disability type.

**Methods:**

This study used nationally representative data from the 2024 Digital Information Divide Survey (DIDS; *N =* 2,155). We employed latent profile analysis to identify digital attitudes–digital self-efficacy profiles and regression models to examine their associations with digital application competence, including tests of disability type moderation while adjusting for demographic, socioeconomic, and disability-related covariates.

**Results:**

First, latent profile analysis identified five profiles: Low Attitudes–Low Self-Efficacy, Moderate Attitudes–Moderate Self-Efficacy, High Attitudes–Low Self-Efficacy, High Attitudes–High Self-Efficacy, and Very High Attitudes–Very High Self-Efficacy. Second, relative to the Low Attitudes–Low Self-Efficacy profile, all other profiles were associated with higher digital application competence, with the largest advantages observed for the High Attitudes–High Self-Efficacy and Very High Attitudes–Very High Self-Efficacy profiles. Finally, disability type did not significantly moderate the association between psychological profiles and competence, although baseline digital application competence differed across disability types.

**Conclusion:**

These findings underscore meaningful psychological heterogeneity among adults with disabilities and show that attitude–self-efficacy configurations are systematically linked to task-oriented digital competence. Digital inclusion efforts should combine profile-informed support, especially strengthening digital self-efficacy, with accessibility-oriented design and structural supports in digital service environments.

## 1 Introduction

With the rapid advancement of information technology, digital technologies have increasingly become essential infrastructure through which individuals access public resources and participate in social life. For people with disabilities, digital technologies have substantial compensatory and empowering potential, helping to reduce barriers associated with physical and mobility constraints and thereby improving overall quality of life (Chemnad and Othman, 2024; Almufareh et al., 2024; Malik et al., 2024). However, the benefits of digitalization are not distributed equally. People with disabilities are more likely to encounter constraints in access, skills, and actual use, resulting in a persistent digital divide (Dobransky and Hargittai, 2016; Pettersson et al., 2023; Park, 2022). In South Korea, people with disabilities remain digitally disadvantaged, and this gap has gradually shifted from disparities in access to disparities in competence and use (Cho and Kim, 2021). Moreover, as social services, including applications for public welfare benefits, social security authentication, telemedicine, and administrative procedures, continue to be digitized (Moon, 2024; Bae et al., 2025), limited digital competence may further restrict access to public services and contribute to the underutilization of institutional resources (Park, 2022; Glumbić et al., 2022). Accordingly, within the institutional context of the accelerating digitalization of public services in South Korea, it is necessary to further examine disparities in task-oriented digital competence among people with disabilities and the mechanisms underlying these disparities.

The shift from access-based inequality to competence- and use-based inequality is consistent with broader developments in digital divide research. Early studies focused primarily on disparities in access, whereas later work expanded the focus to digital skills, patterns of use, and the extent to which online engagement translates into tangible offline benefits (van Deursen and van Dijk, 2011; Glumbić et al., 2022). For people with disabilities, this access-to-outcomes perspective suggests that broad concepts such as digital literacy and digital competence, while useful, are insufficient for capturing digital participation in institutional service contexts. Digital literacy and digital competence have often been used inconsistently in prior research. In general, digital literacy emphasizes foundational knowledge, skills, and critical understanding of digital media, whereas digital competence provides a broader multidimensional framework for describing the knowledge, skills, and attitudes required for confident, critical, and responsible digital technology use (Spante et al., 2018; Tinmaz et al., 2022; Vuorikari et al., 2022). However, neither concept fully captures whether individuals can complete concrete digital tasks in real-world institutional settings. Greater attention is therefore needed to task-oriented performance in such contexts, namely, digital application competence.

In this study, digital application competence refers to an individual’s ability to use digital tools, including mobile devices and computers, to integrate digital resources and complete specific tasks in everyday and institutional settings, thereby reflecting functional, task-oriented performance. This construct should also be distinguished from usability, which refers to the extent to which a system, product, or service can be used by specified users to achieve specified goals with effectiveness, efficiency, and satisfaction in a specified context of use (International Organization for Standardization, 2018). Whereas usability concerns system- or interface-level qualities that shape effective, efficient, and satisfactory use, digital application competence explains whether users can mobilize their skills to complete specific digital tasks within given technological and institutional environments. This conceptual focus aligns with the evolving emphasis in digital divide research, which has shifted from basic access to individuals’ ability to translate digital resources into actionable outcomes. In South Korea’s highly digitalized society, such competence is essential for social inclusion because it shapes individuals’ capacity to navigate instrumental services, including self-service kiosks and mobile public authentication systems.

Regarding the sources of disparities in digital application competence, prior research has emphasized structural and resource-based factors, including access to technology, training opportunities, assistive support, socioeconomic resources, and environmental accessibility (Dobransky and Hargittai, 2016; Khanlou et al., 2021; Cabero-Almenara et al., 2022; Tinmaz et al., 2022; Lazic et al., 2025). Existing studies have also examined digital technology attitudes, digital literacy, and self-efficacy among individuals with disabilities. However, these psychological factors have more often been discussed in relation to general digital inclusion, technology use, technology acceptance, or digital literacy, rather than task-oriented digital application competence in institutional service contexts. This distinction is important because completing concrete digital tasks depends not only on access and support but also on individuals’ confidence, motivation, and orientations toward digital technology. According to social cognitive theory, individuals’ evaluations of digital technology and beliefs about their own capabilities, that is, digital attitudes, and digital self-efficacy, may shape whether they attempt tasks, persist when facing difficulties, and adopt effective coping strategies during real-world digital interactions (Bandura, 1997). Therefore, a specific examination of these psychological factors is necessary to explain the mechanisms underlying disparities in digital application competence among adults with disabilities.

According to social cognitive theory, digital task performance is shaped not only by external resources and environmental conditions but also by individuals’ cognitive-motivational orientations toward technology. Digital attitudes and digital self-efficacy can therefore be understood as complementary psychological determinants. Digital attitudes reflect individuals’ evaluations of the usefulness, value, and relevance of digital technology, which may increase their willingness to engage with digital tools and sustain continued use (Donat et al., 2009; Yu et al., 2025). By contrast, digital self-efficacy refers to individuals’ perceived ability to learn, operate, and master digital devices, thereby influencing effort, persistence, and coping strategies when they encounter digital tasks (Bandura, 1997; Pan, 2020; Rohde et al., 2024). Prior research has shown that both digital attitudes and digital self-efficacy are closely associated with digital literacy and digital engagement (Bentley et al., 2024; Park, 2025; Yu et al., 2025). Moreover, these two factors may reinforce each other: positive digital attitudes may promote participation by strengthening self-efficacy, while stronger self-efficacy may further support digital literacy and favorable technology orientations (Pan, 2020; Getenet et al., 2024; Park, 2025). Conversely, negative attitudes may inhibit digital use and exacerbate digital inequality (Heponiemi et al., 2023). Taken together, digital attitudes and digital self-efficacy may jointly shape whether people with disabilities are both willing and able to translate digital resources into concrete task performance.

However, relatively little research has examined how digital attitudes and digital self-efficacy combine at the person level. Most prior studies have used variable-centered approaches, testing the separate effects or associations. Although useful for identifying average relationships, these approaches cannot fully capture heterogeneity in individuals’ attitude–efficacy configurations. This limitation is important because positive attitudes toward digital technology do not necessarily imply strong confidence in using digital devices; some individuals may recognize the value of digital technologies but still lack the perceived ability to complete digital tasks. Prior work suggests that attitudes and self-efficacy may form distinct psychological engagement patterns that influence digital learning, task practice, and technology acceptance (Yoon and Kim, 2025). Accordingly, examining the joint structure of digital attitudes and digital self-efficacy can provide a more nuanced understanding of psychological heterogeneity in digital application competence among people with disabilities, whose attitudes toward and needs for digital technology may vary substantially (Zhou et al., 2025). Latent profile analysis (LPA) is particularly appropriate for this purpose because it represents a person-centered approach that identifies naturally occurring configurations across multiple continuous indicators without prespecifying group membership (Collins and Lanza, 2013). Its value is not limited to statistical classification. Rather, it provides an epistemological alternative to homogeneous-effect assumptions by allowing digital attitudes and self-efficacy to combine differently across individuals. In this study, LPA enables us to identify distinct psychological configurations among adults with disabilities and to examine whether these configurations are differentially associated with digital application competence.

Further, it is necessary to account for heterogeneity by disability type within South Korea’s population of people with disabilities. According to statistics released by Korea’s Ministry of Health and Welfare, the number of officially registered people with disabilities has reached 2.631 million; by disability-type, physical impairment accounts for the largest share (43.0%), followed by hearing impairment (16.8%), visual impairment (9.4%), brain impairment (8.9%), and intellectual disability (8.9%) (Ministry of Health and Welfare of Korea, 2025). Beyond these compositional differences, disability type can be understood as a functional-context factor because different disability types involve distinct sensory, cognitive, communicative, and operational conditions that may alter the demands and costs of completing digital tasks (Giakoumis et al., 2014; Berget and MacFarlane, 2020). For instance, visual impairment may increase difficulties with interface recognition and information retrieval; hearing or speech impairment may affect digital communication and service interactions; and brain impairment may increase cognitive load when navigating complex interfaces. These differences may shape baseline levels of digital application competence and influence how psychological profiles translate into digital outcomes (Sahib et al., 2012; Moreno et al., 2024; Pettersson et al., 2023). Accordingly, this study examines whether disability type moderates the association between attitude–self-efficacy profiles and digital application competence.

Building on the above discussion, this study aims to: (1) identify latent attitude–self-efficacy profiles among adults with disabilities; (2) examine the associations between these profiles and digital application competence; and (3) test whether disability type moderates the relationship between profile membership and digital application competence. By shifting attention from access-based explanations of the digital divide to psychological heterogeneity and configurational patterns, this study advances understanding of why adults with disabilities differ in their ability to translate digital resources into concrete task performance. The findings provide more fine-grained empirical evidence for explaining within-group disparities in digital competence and offer implications for designing targeted support strategies amid the ongoing digitalization of public services.

## 2 Methods

### 2.1 Data source

This study uses data from the 2024 Digital Information Divide Survey (DIDS), jointly conducted by Korea’s Ministry of Science and ICT (MSIT) and the National Information Society Agency (NIA, 2025). The DIDS is an official annual government statistical survey that assesses the status of the digital information divide and provides an empirical basis for policy design and evaluation. The 2024 DIDS targeted people with disabilities nationwide who were officially registered under the Disability Welfare Act as of August 1, 2024, and were aged 7–69. The sample comprised 2,200 respondents across four disability categories: physical/mobility, brain injury, hearing/speech, and visual impairment. A stratified proportional sampling design was used, with strata defined by gender, age, disability type, and region. Data were collected through structured face-to-face household interviews conducted from October to December 2024, and the results were released in March 2025. For the present study, we restricted the sample to adults. Under Korea’s Youth Protection Act, individuals aged 19 and older are classified as adults; thus, after excluding 45 respondents younger than 19, the final analytic sample comprised 2,155 adults.

### 2.2 Measures

#### 2.2.1 Dependent variable

The dependent variable was digital application competence. In the 2024 DIDS, this construct corresponds to the survey’s information competence domain, integrated across computers/PCs and smart devices. It was operationalized using nine self-reported task items that captured respondents’ perceived ability to perform common digital activities on mobile phones and computers. Specifically, the items assessed the perceived ability to use functional applications (e.g., calculator, scheduler, contacts), participate in remote meetings, operate smart devices (including IoT), edit and format digital content, collaborate online for work tasks, make mobile payments, use maps and transportation information, use self-service kiosks (e.g., ordering food, purchasing tickets, checking in at hospitals), and complete electronic authentication. Items were rated on a 4-point scale (1 = not at all capable to 4 = very capable) and summed to create a total score ranging from 9 to 36, with higher scores indicating greater digital application competence. In this study, the scale showed high internal consistency (Cronbach’s *α* = 0.94).

#### 2.2.2 Independent variable

The independent variables were digital attitudes and digital self-efficacy. These constructs and corresponding self-report items have been widely used in prior research to capture individuals’ appraisals of digital technology and confidence in learning and using digital devices (Park, 2025; Yu et al., 2025). Digital attitudes represent an overall evaluation of digital technology and were assessed with four items reflecting perceived usefulness (“Digital technologies are useful”), perceived convenience (“Digital technologies make my life more convenient”), perceived personal value (“Digital technologies are beneficial to me”), and intention to increase use (“I want to use digital technologies more frequently”). Digital self-efficacy refers to perceived competence in learning and using digital devices and was assessed with four items capturing learning efficacy (“I am confident in my ability to learn how to use digital devices”), operational efficacy (“I am confident in my ability to use digital devices”), mastery efficacy (“I can quickly master the use of new digital devices”), and intention to increase use (“I want to use digital devices more frequently”). All eight items were rated on a 4-point Likert scale (1 = strongly disagree to 4 = strongly agree). Scale scores were computed as the mean of the four items for each construct, with higher scores indicating more positive digital attitudes and greater digital self-efficacy. In this study, internal consistency was good for digital attitudes (Cronbach’s *α* = 0.87) and digital self-efficacy (Cronbach’s α = 0.89). Consistent with the 2024 DIDS survey design, digital attitudes and self-efficacy were assessed as general psychological orientations toward digital technology rather than being tied to specific devices. While this approach captures overarching cognitive-motivational appraisals, it is acknowledged that respondents’ evaluations may reflect their cumulative experiences across various device environments. Using the eight item-level indicators, we conducted latent profile analysis to identify distinct attitude–self-efficacy profiles.

#### 2.2.3 Moderator variable

Disability type was examined as a moderator and defined according to Korea’s official disability registration system. In Korea, disability types are legally defined within the Korea National Disability Registration System (KNDRS), which classifies disabilities into multiple legally specified categories through standardized medical assessment procedures (Kim et al., 2023). In the 2024 DIDS, disability type was grouped into four categories: physical/mobility, brain impairment, hearing/speech impairment, and visual impairment, consistent with the major functional domains used in the national registration framework.

#### 2.2.4 Covariates

Following prior research (Cho and Kim, 2021; Park, 2022; Yu et al., 2025), seven variables were included as covariates: age, gender, place of residence, education, household income, onset of disability, and severity of disability. Coding was as follows: age was treated as a continuous variable; gender was coded as 0 = female and 1 = male; place of residence was coded as 0 = rural (myeon; township-level areas) and 1 = urban (dong; neighborhood-level areas); education was classified into four categories (0 = primary school or below, 1 = middle school, 2 = high school, 3 = university or above) and treated as an ordinal variable; household income was measured in 11 ordered categories (1 = monthly income < 1 million KRW; 11 = ≥ 10 million KRW) and treated as approximately continuous; onset of disability was coded as 0 = acquired and 1 = congenital; and severity of disability coded as mild versus severe, reflecting the current KNDRS classification, in which grades 1–3 are recognized as severe disabilities and grades 4–6 as mild disabilities (Kim et al., 2023).

### 2.3 Data analysis

First, sample characteristics were summarized using means (M) and standard deviations (SD) for continuous variables and frequencies (N) and percentages (%) for categorical variables. Second, LPA was conducted using the eight indicators of digital attitudes and digital self-efficacy to identify distinct attitude–self-efficacy profiles. Third, analysis of variance (ANOVA) was used for continuous variables, and Pearson’s *χ*^2^ tests were used for categorical variables to examine differences in demographic and disability-related characteristics across profiles. Fourth, multinomial logistic regression was conducted to examine demographic, socioeconomic, and disability-related correlates of profile membership. Finally, linear regression models were estimated to examine the association between profile membership and digital application competence, with interaction terms between profile membership and disability type included to test moderating effects. LPA was conducted using Mplus 8.3, while all other analyses were performed using Stata 18.0. A *p <* 0.05 was considered indicative of statistical significance.

To evaluate model fit and select the appropriate number of latent profiles, we considered multiple statistical criteria. First, we compared the Akaike information criterion (AIC), Bayesian information criterion (BIC), and sample-size–adjusted BIC (aBIC) across models; lower values indicate better relative fit among competing models (Nylund et al., 2007). Second, we used the Lo–Mendell–Rubin (LMR) and bootstrap likelihood ratio test (BLRT) to compare adjacent models; *p <* 0.05 indicates that the model with k profiles fits significantly better than the model with k – 1 profiles (Nylund et al., 2007). Third, we examined entropy as an index of classification accuracy, with values closer to 1.0 indicating clearer classification (Lubke and Muthén, 2007). Finally, we evaluated profile proportions to ensure interpretability and practical relevance; as a rule of thumb, profiles comprising more than 5% of the sample are often typically considered more stable and interpretable.

## 3 Results

### 3.1 Sample characteristics by disability type

Table 1 presents descriptive statistics for the study sample and tests for differences across disability types. The analytic sample included 2,155 adults with disabilities, with a mean age of 52.10 years (SD = 13.09). Men comprised 68.63% of the sample, and most respondents lived in urban areas (87.29%). Regarding education, 54.57% of participants had completed high school. The most common disability type was physical impairment (59.54%); 72.16% of respondents reported an acquired disability, and 62.18% were classified as having mild disability severity. For the key study variables, the mean score for digital application competence was 21.99 (SD = 7.07), the mean digital attitudes score was 11.66 (SD = 2.53), and the mean digital self-efficacy score was 9.78 (SD = 2.90). Except for place of residence, all variables differed significantly across disability types (*p <* 0.05).

**Table 1:** Sample characteristics and differences across disability types (*N =* 2,155).

Variables	Total	PI	VI	BI	HIS	F/*χ*^2^
	2,155 (100)	1,283	308	286	278	
Age (19–69)	52.10 (13.09)	53.89 (11.56)	48.53 (13.78)	48.58 (15.37)	51.45 (14.78)	23.44^***^
Gender						
Male	1,479 (68.63)	930 (72.49)	200 (64.94)	185 (64.69)	164 (58.99)	24.88^***^
Female	676 (31.37)	353 (27.51)	108 (35.06)	101 (35.31)	114 (41.01)	
Residence						
Urban	1,881 (87.29)	1,132 (88.23)	269 (87.34)	243 (84.97)	237 (85.25)	3.46
Rural	274 (12.71)	151 (11.77)	39 (12.66)	43 (15.03)	41 (14.75)	
Education						
Primary school or below	118 (5.48)	62 (4.83)	7 (2.27)	24 (8.39)	25 (8.99)	57.47^***^
Middle school	369 (17.12)	208 (16.21)	39 (12.66)	57 (19.93)	65 (23.38)	
High school	1,176 (54.57)	725 (56.51)	156 (50.65)	153 (53.50)	142 (51.08)	
University or above	492 (22.83)	288 (22.45)	106 (34.42)	52 (18.18)	46 (16.55)	
Onset of disability						
Congenital	600 (27.84)	289 (22.53)	81 (26.30)	114 (39.86)	116 (58.27)	65.65^***^
Acquired	1,555 (72.16)	994 (77.47)	227 (73.70)	172 (60.14)	162 (58.27)	
Severity of disability						
Mid	1,340 (62.18)	828 (64.54)	212 (68.83)	136 (47.55)	164 (58.99)	36.05^***^
Severe	815 (37.82)	455 (35.46)	96 (31.17)	150 (52.45)	114 (41.01)	
Income (1–11)	3.82 (1.91)	3.86 (1.91)	4.13 (2.00)	3.53 (1.91)	3.56 (1.77)	6.83^***^
DAC (9–36)	21.99 (7.07)	22.60 (6.70)	22.40 (7.53)	19.90 (7.12)	20.90 (7.62)	14.32^***^
DA (4–16)	11.66 (2.53)	11.71 (2.43)	12.03 (2.48)	11.14 (2.64)	11.58 (2.82)	6.66^***^
DSE (4–16)	9.78 (2.90)	9.92 (2.80)	10.18 (2.85)	9.00 (2.97)	9.52 (3.15)	10.81^***^

^*^p < 0.05, ^**^p < 0.01, ^***^p < 0.001; the values represent the sample sizes (%) or sample means (SD). SD, Standard Deviation; PI, Physical Impairment; VI, Visual Impairment; BI, Brain Impairment; HSI, Hearing/speech Impairment; DAC, Digital Application Competence; DA, Digital Attitudes; DSE, Digital Self-Efficacy.

### 3.2 Latent profile analysis of digital attitudes and digital self-efficacy

Table 2 presents the fit statistics for the latent profile models. From the two- to five-profile solutions, AIC, BIC, and aBIC decreased monotonically, suggesting improved relative fit as additional profiles were extracted. The BLRT was significant for all estimable models (*p <* 0.001), and the LMR test supported the five-profile solution over the four-profile solution (*p* = 0.0099). Although the six-profile solution was examined, it was rejected because the model did not terminate normally and was not stable across random starts. Entropy for the five-profile solution was modest (0.688), suggesting that classification uncertainty should be considered when interpreting the profiles. Nevertheless, the average posterior probabilities were high (0.909–0.963; see Supplementary Table S1), and the five-profile solution was theoretically interpretable. Taken together, these findings supported the selection of the five-profile solution.

**Table 2:** Fit indices for latent profile models.

Profile	AIC	BIC	aBIC	Entropy	LMR (P)	BLRT (P)	Class probabilities
2c	34978.98	35120.87	35041.44	0.85	0.0015	0.0000	0.4190/0.5810
3c	32998.39	33191.36	33083.34	0.87	0.0034	0.0000	0.1197/0.4241/0.4562
4c	31806.67	32050.72	31914.10	0.88	0.0002	0.0000	0.0868/0.3917/0.4278/0.0937
5c	30710.80	31005.93	30840.72	0.89	0.0099	0.0000	0.0840/0.1921/0.2492/0.3810/0.0937
6c	–	–	–	0.688	–	–	0.0566/0.2432/0.1485/0.1759/0.3183/0.0575

AIC, Akaike Information Criterion; BIC, Bayesian Information Criterion; aBIC, Adjusted Bayesian Information Criterion; LMR, Lo–Mendell–Rubin; BLRT, Bootstrapped Likelihood Ratio Test.

**Figure 1:**
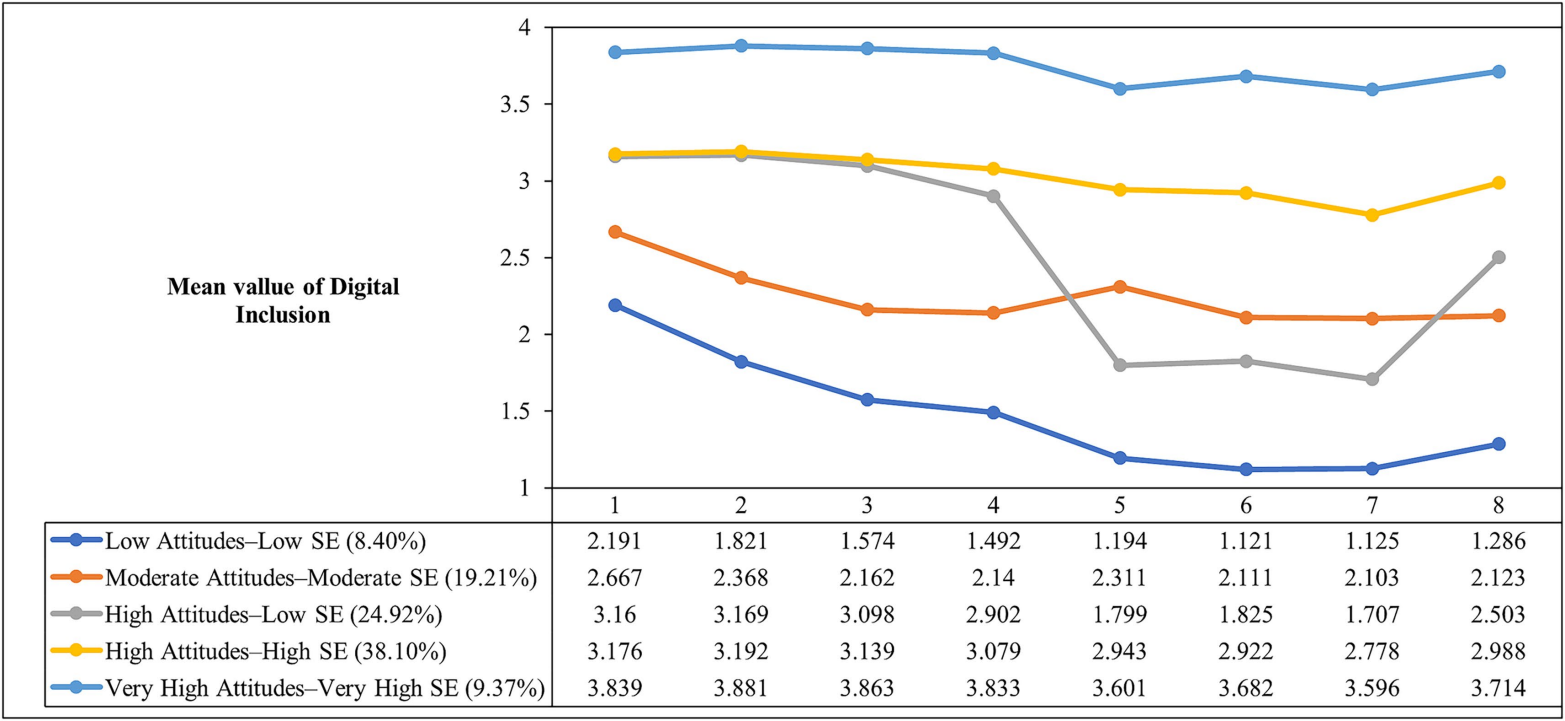
Latent profile analysis of digital attitudes and digital self-efficacy. (1) Perceived usefulness of digital technology, (2) perceived convenience of digital technology, (3) perceived personal value of digital technology, and (4) intention to increase use of digital technologies. (5) Learning efficacy for digital devices, (6) operational efficacy for digital devices, (7) mastery efficacy for new digital devices, (8) intention to increase use of digital devices. SE, Self-efficacy.

Figure 1 displays the latent profiles derived from the LPA based on the mean item scores of the eight indicators. Items 1–4 assessed digital attitudes, and Items 5–8 assessed digital self-efficacy. Higher scores indicate more positive evaluations of digital technology and greater confidence in learning and using digital devices. Overall, the profiles show discernible differences across both the attitude and self-efficacy domains, indicating meaningful heterogeneity in attitude–efficacy configurations among adults with disabilities.

Specifically, Profile 1 was characterized by consistently low scores across all eight indicators and comprised 8.40% of the sample; it was labeled the “Low Attitudes–Low Self-Efficacy”. Profile 2 displayed mid-range scores on both the attitude and self-efficacy domains and accounted for 19.21% of the sample; it was labeled the “Moderate Attitudes–Moderate Self-Efficacy”. Profile 3 showed relatively high scores on the attitude items but markedly lower scores on the self-efficacy items, indicating an attitude–self-efficacy discrepancy, and comprised 24.92% of the sample; it was labeled the “High Attitudes–Low Self-Efficacy”. Profile 4 exhibited high scores in both domains, though generally lower than those in profile 5, and represented 38.10% of the sample; it was labeled the “High Attitudes–High Self-Efficacy”. Profile 5 demonstrated the highest scores across all indicators and comprised 9.37% of the sample; it was labeled the “Very High Attitudes–Very High Self-Efficacy”. Taken together, these profiles reflect a progression from low to very digital attitudes and self-efficacy, with profile 3 uniquely capturing a misalignment between attitudes and perceived self-efficacy.

### 3.3 Sample characteristic differences across digital attitude–digital self-efficacy profiles

Table 3 shows that sample characteristics varied across the five attitude–self-efficacy profiles. The Low Attitudes–Low Self-Efficacy profile was composed primarily of older individuals with lower educational attainment and lower household income, and it also showed the highest proportion of severe disability. The Moderate Attitudes–Moderate Self-Efficacy profile displayed a mid-range distribution of age and socioeconomic resources, with many respondents concentrated at the high-school education level. The High Attitudes–Low Self-Efficacy profile was characterized by relatively positive attitudes but comparatively lower self-efficacy; in terms of sample composition, respondents were more often older and had lower educational attainment than those in the High Attitudes–High Self-Efficacy and Very High Attitudes–Very High Self-Efficacy profiles. The High Attitudes–High Self-Efficacy profile included a larger share of younger and more educated respondents and was more socioeconomically advantaged overall. The Very High Attitudes–Very High Self-Efficacy profile showed the most favorable pattern, with respondents more often younger and university-educated, and having the highest level of digital application competence.

**Table 3:** Characteristic differences in digital attitude–digital self-efficacy profiles.

Variables	Low attitudes–low SE (181, 8.40%)	Moderate attitudes–moderate SE (414, 19.21%)	High attitudes–low SE (537, 24.92%)	High attitudes–high SE (821, 38.10%)	Very high attitudes–very high SE (202, 9.37%)	F/*χ*^2^
Age	58.38 (12.17)	55.90 (11.78)	56.55 (11.78)	48.18 (12.31)	42.82 (12.81)	91.55^***^
Gender						
Male	109 (60.22)	291 (70.29)	344 (64.06)	584 (71.13)	151 (74.75)	17.59^**^
Female	72 (39.78)	123 (29.71)	193 (35.94)	237 (28.87)	51 (25.25)	
Residence						
Urban	151 (83.43)	384 (92.75)	454 (84.54)	715 (87.09)	177 (87.62)	17.27^**^
Rural	30 (16.57)	30 (7.25)	83 (15.46)	106 (12.91)	25 (12.38)	
Education						
Primary school or below	38 (20.99)	24 (5.80)	45 (8.38)	7 (0.85)	4 (1.98)	527.72^***^
Middle school	70 (38.67)	88 (21.26)	135 (25.14)	67 (8.16)	9 (4.46)	
High school	72 (39.78)	256 (61.84)	308 (57.36)	462 (56.27)	78 (38.61)	
University or above	1 (0.55)	46 (11.11)	49 (9.12)	285 (34.71)	111 (54.95)	
Disability type						
PI	92 (50.83)	244 (58.94)	314 (58.47)	516 (62.85)	117 (50.92)	39.83^***^
VI	17 (9.39)	60 (14.49)	68 (12.66)	124 (15.10)	39 (19.31)	
BI	38 (20.99)	59 (14.25)	87 (16.20)	86 (10.48)	16 (7.92)	
HSI	34 (18.78)	51 (12.32)	68 (12.66)	95 (11.57)	30 (14.85)	
Onset of disability						
Congenital	74 (40.88)	126 (30.43)	144 (26.82)	218 (26.55)	38 (18.81)	25.87^***^
Acquired	107 (59.12)	288 (69.57)	393 (73.18)	603 (73.45)	164 (81.19)	
Severity of disability						
Mid	90 (49.72)	232 (56.04)	296 (55.12)	600 (73.08)	122 (60.40)	71.72^***^
Severe	91 (50.28)	182 (43.96)	241 (44.88)	221 (26.92)	80 (39.60)	
Income	2.66 (1.33)	3.66 (1.88)	3.21 (1.67)	4.42 (1.83)	4.34 (2.30)	63.64^***^
DAC	11.86 (3.95)	19.74 (5.93)	18.56 (5.15)	25.64 (4.76)	29.98 (5.80)	55.17^***^

^*^p < 0.05, ^**^ *p <* 0.01, ^***^ *p <* 0.001; the values represent the sample sizes (%) or sample means (SD). SD, Standard Deviation; PI, Physical Impairment; VI, Visual Impairment; BI, Brain Impairment; HSI, Hearing/speech Impairment; DAC, Digital Application Competence; SE, Self-Efficacy.

In addition, disability type distributions differed across profiles (*χ*^2^ = 39.83, *p <* 0.001). Physical impairment accounted for the largest share in every profile. Brain impairment was relatively more concentrated in the Low Attitudes–Low Self-Efficacy profile (20.99%). Visual impairment showed the opposite pattern, with a higher proportion in the Very High Attitudes–Very High Self-Efficacy profile (19.31%) than in the Low Attitudes–Low Self-Efficacy profile (9.39%). Hearing/speech impairment varied more modestly across profiles, ranging from 11.57 to 18.78%.

### 3.4 Associations between digital attitude-digital self-efficacy profiles, disability types, and digital application competence

Before examining the association between the digital attitude–digital self-efficacy profiles and digital application competence, we estimated multinomial logistic regression models to identify significant predictors of profile membership (reference: High Attitudes–High Self-Efficacy). Significant predictors included age, educational attainment, and disability-related characteristics (i.e., disability type, onset of disability, and disability severity); detailed results are reported in Supplementary Table S2.

Table 4 presents the regression results for digital application competence (overall model). Using the Low Attitudes–Low Self-Efficacy profile as the reference group, membership in all other profiles was positively associated with digital application competence, including the Moderate Attitudes–Moderate Self-Efficacy profile (*β* = 0.33, *p <* 0.001), the High Attitudes–Low Self-Efficacy profile (*β* = 0.33, *p <* 0.001), the High Attitudes–High Self-Efficacy profile (*β* = 0.69, *p <* 0.001), and the Very High Attitudes–Very High Self-Efficacy profile (*β* = 0.58, *p <* 0.001). Among covariates, age (*β* = −0.11, *p <* 0.001) and disability severity (*β* = −0.08, *p <* 0.001) were negatively associated with competence, whereas education (middle school: *β* = 0.10, *p <* 0.001; high school: *β* = 0.27, *p <* 0.001; university or above: *β* = 0.31, *p <* 0.001), congenital onset (*β* = 0.03, *p <* 0.05), and income (*β* = 0.15, *p <* 0.001) were positively associated with competence. Gender and place of residence were not significant. Relative to physical impairment, visual impairment (*β* = −0.07, *p <* 0.001), brain impairment (*β* = −0.06, *p <* 0.001), and hearing/speech impairment (*β* = −0.04, *p <* 0.01) were associated with lower competence scores. The model explained a substantial proportion of variance (R^2^ = 0.58; *F* = 184.65, *p <* 0.001).

**Table 4:** Association between latent profiles of digital attitudes–self-efficacy and digital application competence (overall and classified by disability types).

Variables	Model 1	Model 2	Model 3	Model 4	Model 5
	Overall	PI	VI	BI	HSI
Profile (ref. Low Attitudes–Low SE)					
Moderate Attitudes–Moderate SE	0.33^***^ (0.06)	0.35^***^ (0.07)	0.35^***^ (0.20)	0.41^***^ (0.15)	0.21^***^ (0.17)
High Attitudes–Low SE	0.33^***^ (0.06)	0.36^***^ (0.07)	0.36^***^ (0.20)	0.34^***^ (0.14)	0.22^**^ (0.16)
High Attitudes–High SE	0.69^***^ (0.06)	0.72^***^ (0.07)	0.65^***^ (0.19)	0.72^***^ (0.15)	0.56^***^ (0.16)
Very High Attitudes–Very High SE	0.58^***^ (0.07)	0.60^***^ (0.09)	0.63^***^ (0.22)	0.48^***^ (0.22)	0.48^***^ (0.20)
Age	−0.11^***^ (0.02)	−0.15^***^ (0.02)	−0.12^**^ (0.04)	−0.02 (0.04)	−0.05 (0.05)
Male (ref. Female)	0.02 (0.03)	0.02 (0.04)	0.00 (0.09)	0.01 (0.09)	−0.02 (0.09)
Education (ref. Primary school or below)					
Middle school	0.10^***^ (0.07)	0.10^**^ (0.09)	0.09 (0.30)	0.05 (0.18)	0.12 (0.17)
High school	0.27^***^ (0.07)	0.31^***^ (0.08)	0.07 (0.29)	0.20^*^ (0.17)	0.34^***^ (0.18)
University or above	0.31^***^ (0.08)	0.34^***^ (0.10)	0.19 (0.30)	0.19^*^ (0.20)	0.37^***^ (0.22)
Income	0.15^***^ (0.02)	0.15^***^ (0.02)	0.21^***^ (0.04)	0.16^**^ (0.05)	0.10^*^ (0.06)
Urban (ref. Rural)	−0.01(0.04)	−0.02(0.05)	0.04(0.12)	−0.05(0.12)	0.01(0.13)
Disability type (ref. PI)					
VI	−0.07^***^(0.04)				
BI	−0.06^***^(0.04)				
HSI	−0.04^**^(0.04)				
Congenital (ref. Acquired)	0.03^*^ (0.03)	0.02 (0.04)	0.09^*^ (0.10)	0.09^*^ (0.10)	0.03 (0.10)
Severity (ref. Not severe)	−0.08^***^ (0.03)	−0.02 (0.04)	−0.29^***^ (0.09)	−0.12^**^ (0.09)	−0.10^*^ (0.10)
R-squared	0.5802	0.6116	0.5744	0.5534	0.5745
Adj R-squared	0.5770	0.6076	0.5556	0.5320	0.5536
F	184.65^***^	153.73^***^	30.53^***^	25.92^***^	27.42^***^

^*^p < 0.05, ^**^p < 0.01, ^***^p < 0.001; PI, Physical Impairment; VI, Visual Impairment; BI, Brain Impairment; HSI, Hearing/speech Impairment. Standardized coefficients (*β*) are reported, with their standard errors in parentheses. SE, Self-Efficacy.

To test whether disability type moderates the association between profile membership and digital application competence, we added interaction terms between disability type and profile membership. The interaction terms were jointly non-significant [*F*(12, 2,126) = 0.93, *p* = 0.513], indicating no evidence that disability type moderates this association. We also conducted an omnibus joint test of the disability-type main effects and the interaction terms [*F*(15, 2,126) = 3.16, *p <* 0.0001]. Combined with the margin results, which show that predicted competence increases across profiles for all disability types and that the lines are approximately parallel with largely overlapping confidence intervals, this pattern suggests that between-group differences are driven primarily by baseline differences in digital application competence across disability types rather than by heterogeneous profile effects. For presentation and interpretability, we additionally report disability-type–stratified regression results (Table 4) to describe whether the profile–competence associations are generally consistent across disability types. The stratified OLS models show that, relative to the Low Attitudes–Low Self-Efficacy profile, all other profiles are associated with higher digital application competence across disability types.

## 4 Discussion

This study used data from the 2024 Digital Information Divide Survey (DIDS) to examine digital attitudes and digital self-efficacy among adults with disabilities. Using latent profile analysis, we identified joint psychological profiles and assessed their associations with digital application competence, and tested whether disability type moderated these associations. The findings show that: (1) digital attitudes and digital self-efficacy co-occurred in heterogeneous ways, yielding five distinct attitude–self-efficacy profiles; (2) relative to the Low Attitudes–Low Self-Efficacy profile, all other psychological profiles were significantly associated with higher digital application competence; and (3) the overall moderating effect of disability type on the association between psychological profiles and digital application competence was not statistically significant, although disability type was associated with baseline differences in digital application competence and descriptive subgroup patterns varied to some extent. Overall, this study demonstrates heterogeneity in psychological configurations within the disability population and clarifies how these configurations relate to competence in task-oriented digital applications.

Overall, the five profiles exhibited a stratified pattern in digital attitudes and digital self-efficacy, ranging from low to high, while also revealing a key asymmetric configuration: the High Attitudes–Low Self-Efficacy profile. This profile indicates that some adults with disabilities positively evaluate the value of digital technologies and express a relatively strong willingness to use them, yet lack sufficient confidence in their ability to learn, operate, and master digital devices. Rather than reflecting a simple “willing versus unwilling” distinction based on a single attitude indicator, this pattern is more consistent with a psychological state characterized by high willingness but insufficient efficacy beliefs.

In terms of sample composition, the High Attitudes–Low Self-Efficacy profile accounted for a substantial share of the sample (24.92%). Individuals in this profile tended to be older and have relatively lower educational attainment, with a higher concentration of respondents with brain impairment and severe disabilities. Their digital application competence was higher than that of the Low Attitudes–Low Self-Efficacy profile, but clearly lower than that of profiles with high self-efficacy. These findings suggest that digital competence development may be shaped by compounding disadvantages, as older age, lower educational attainment, and severe disability do not operate in isolation. Limited educational resources and age-related challenges may be further intensified by functional impairments, contributing to the restrictive attitude–efficacy configuration observed in this profile. This pattern may limit the translation of willingness to use digital technologies into stable task performance, suggesting that the primary constraint for this group lies less in value recognition than in limited efficacy beliefs during operational processes. Although prior research indicates that digital attitudes and self-efficacy are closely related to digital outcomes (Park, 2025; Heponiemi et al., 2023; Getenet et al., 2024; Cho and Kim, 2021), the present study shows that these psychological factors do not always increase in tandem, providing fine-grained evidence of within-group differentiation. Future research should move beyond additive models to formally test how these determinants interact within larger, targeted samples.

This study found that, relative to the Low Attitudes–Low Self-Efficacy profile, all other profiles were associated with higher digital application competence, with the largest advantages observed for the High Attitudes–High Self-Efficacy and Very High Attitudes–Very High Self-Efficacy profiles. This pattern is consistent with social cognitive theory, which emphasizes that behavioral performance is shaped not only by external conditions but also by individuals’ belief systems, particularly self-efficacy (Bandura, 1997). In task-oriented digital contexts, positive attitudes may increase willingness to engage, whereas strong self-efficacy may support effort, persistence, and coping when difficulties arise. Notably, the High Attitudes–Low Self-Efficacy profile showed only an intermediate level of competence, suggesting that positive attitudes alone may not translate into effective task performance when efficacy beliefs are weak. This finding points to a psychological digital barrier in the second-level digital divide, whereby willingness to use technology is not fully translated into task performance due to insufficient confidence in one’s ability to complete digital tasks. Importantly, these psychological profiles should not be interpreted as purely internal traits or individual preferences; rather, they may also reflect unequal opportunities for digital exposure and practice shaped by age, education, disability severity, and functional constraints. In this sense, attitude–self-efficacy profiles represent a mechanism by which external inequality translates into internal constraints on motivation, confidence, and task performance.

Regarding disability type, this study found no statistically significant overall moderating effect on the association between membership in the psychological profile and digital application competence. This suggests that the relationship between attitude–self-efficacy profiles and digital application competence was broadly consistent across disability groups: relative to the Low Attitudes–Low Self-Efficacy profile, the other profiles were generally associated with higher competence. At the same time, significant omnibus tests for disability-type–related terms indicate that mean levels of digital application competence differed across disability categories. Thus, disability type appears to shape baseline competence levels rather than fundamentally altering the psychological mechanism linking profiles to competence. This interpretation is consistent with prior research suggesting that disability-type differences in digital competence may vary across task contexts and measurement approaches (Cabero-Almenara et al., 2022). Overall, disability-type differences may reflect variation in external constraints, interaction costs, and accessibility conditions that shape baseline performance (Dobransky and Hargittai, 2016; Pettersson et al., 2023; Vicente and López, 2010), whereas the benefits associated with stronger digital attitudes and self-efficacy appear directionally similar across groups. Future research should further examine whether disability type indirectly influences digital application competence by shaping prior exposure to digital devices, assistive technologies, and opportunities for practice, which may in turn affect the development of digital attitudes and self-efficacy.

In terms of profile membership, the multinomial logit results further indicate that older age and lower socioeconomic resources were associated with a higher likelihood of belonging to less favorable profiles, such as Low Attitudes–Low Self-Efficacy and High Attitudes–Low Self-Efficacy. Respondents with brain impairment, congenital onset, and severe disability were also more likely to fall into these less favorable profiles. At the same time, age, educational attainment, household income, and disability severity were significantly associated with digital application competence: older age and greater disability severity were associated with lower competence, whereas higher education and income were associated with higher competence. This pattern is consistent with digital divide research emphasizing inequalities in resource availability and opportunities for competence development (Dobransky and Hargittai, 2016; Pettersson et al., 2023) and aligns with Park (2025), suggesting that structural conditions such as education and income operate alongside psychological factors to shape digital outcomes. Taken together, these findings support a more integrative interpretation: psychological configurations do not emerge independently of structural conditions, and resources and functional constraints may shape attitudes and efficacy beliefs by affecting opportunities for digital exposure, the costs of practice, and the accumulation of failure experiences. This perspective also helps explain why, in the institutional context of the ongoing digitalization of public services, some groups express relatively strong willingness to use digital technologies but insufficient efficacy beliefs.

These findings have theoretical, practical, and policy implications. Theoretically, they challenge a purely additive understanding of psychological determinants in digital divide research. If digital attitudes and self-efficacy were considered only as separate linear predictors, individuals with positive attitudes would be expected to exhibit consistently high levels of digital application competence. However, the High Attitudes–Low Self-Efficacy profile demonstrates that positive evaluations of digital technology do not necessarily translate into task-oriented competence when perceived capability remains weak. This finding supports a configurational understanding of digital competence, in which the alignment or mismatch between motivational readiness and perceived capability shapes whether adults with disabilities can convert digital resources into concrete task performance.

Practically, profile-informed interventions should be tailored to distinct attitude–self-efficacy configurations, with particular attention to the High Attitudes–Low Self-Efficacy profile. This profile is important because it reveals that digital exclusion does not always arise from resistance to technology or lack of perceived value. For a substantial subgroup of adults with disabilities, the key barrier may instead lie in insufficient efficacy beliefs that prevent willingness from being translated into stable task performance. Therefore, digital inclusion strategies should not only promote positive attitudes but also strengthen mastery experiences and confidence through repeated, accessible, and task-specific support. These supports should follow scaffolded, mastery-based learning pathways in which complex digital tasks are divided into manageable steps, repeated successful experiences are accumulated, and timely feedback reinforces self-efficacy during task completion. For individuals in the Low Attitudes–Low Self-Efficacy and Moderate Attitudes–Moderate Self-Efficacy profiles, low-threshold, scenario-based demonstrations and guided practice in low-risk settings may strengthen perceived value and gradually enhance digital self-efficacy. For the High Attitudes–Low Self-Efficacy profile, interventions should focus more directly on self-efficacy building by breaking digital tasks into manageable steps, providing repeated practice opportunities, offering timely feedback, and delivering individualized guidance to reduce uncertainty in learning and operation. Evidence from a randomized controlled trial indicates that digital self-efficacy training can yield beneficial effects (Rohde et al., 2024), supporting the value of confidence-oriented interventions amid the ongoing digitalization of public services.

From a policy perspective, the non-significant moderating effect of disability type suggests that disability-type differences should be addressed primarily by raising baseline digital competence and reducing accessibility barriers, rather than by assuming fundamentally different psychological mechanisms across disability groups. Disability type appears to shape baseline competence through functional demands, accessibility conditions, and interaction costs, while the benefits associated with more favorable attitude–self-efficacy profiles remain broadly similar across groups. Accordingly, digital public services should combine universal design with targeted accommodations that address the specific barriers faced by different disability groups (Berget and MacFarlane, 2020). For individuals with visual impairments, this includes strengthening screen-reader compatibility, semantic labeling, and accessible authentication procedures, as well as embedding assistive technologies into task-based training in real-world service scenarios (Belachew et al., 2025). For individuals with cognitive or executive-function limitations, simplifying interfaces, reducing procedural steps, and providing stable entry points may lower cognitive load and implementation burden (Moreno et al., 2024; Vereenooghe et al., 2021). Artificial intelligence may further support disability inclusion through accessible interaction, assisted communication, personalized task guidance, and self-efficacy feedback, but its implementation should follow accessibility-by-design principles and appropriate ethical and governance frameworks (Almufareh et al., 2024).

There are several limitations to this study. First, because the analyses relied on cross-sectional data, the associations between psychological profiles and digital application competence should not be interpreted causally. Longitudinal or intervention studies are needed to establish temporal ordering and assess causal pathways. Second, although this study focused on digital attitudes and digital self-efficacy, it did not directly measure structural conditions such as institutional accessibility, service availability, platform and interface design, or the assistive technology environment. Future research should incorporate these contextual factors to better explain how structural conditions shape digital application competence. Third, the analytic sample was limited to registered adults with disabilities who were able to complete face-to-face household interviews, which may underestimate digital disadvantage among individuals with more severe functional limitations or those living in hard-to-reach areas. Fourth, the measures of digital attitudes and digital self-efficacy referred to digital technologies or devices in general and did not distinguish among smartphones, computers, self-service kiosks, authentication systems, or assistive technologies. Because disability groups may differ in device-specific access and use experiences, future research should examine whether such experiences mediate the associations among disability type, psychological beliefs, and digital application competence. Fifth, the latent profiles may be sample-dependent, and the self-reported indicators may involve indicator dependency and response bias. Future studies should validate the profile structure using independent samples, alternative indicators, external validation variables, and behavioral or task-based assessments of digital competence.

## 5 Conclusion

This study used national survey data to identify five distinct attitude–self-efficacy profiles among Korean adults with disabilities. Profile membership significantly predicted digital application competence, and disability type was associated with differences in both profile membership and digital application competence. However, disability type did not significantly moderate the association between profile membership and digital application competence, suggesting that the role of psychological profiles was broadly similar across disability groups. This finding indicates that even among disability groups with lower baseline competence, tailored support to strengthen digital self-efficacy may help improve task-oriented digital competence. These findings extend research on digital inequality by linking person-centered psychological configurations to task-oriented digital outcomes. Digital inclusion initiatives should therefore combine profile-informed psychological support with accessibility-oriented technology design, structural supports, and inclusive policies. In this sense, digital inclusion should be understood not only as access equity but also as competence-enabled use equity, through which adults with disabilities are supported in transforming digital access into confident, effective, and meaningful use.

## Data Availability

Publicly available datasets were analyzed in this study. This data can be found at: https://www.data.go.kr/data/15038422/fileData.do.

## References

[ref1] Almufareh M. F. Kausar S. Humayun M. Tehsin S. (2024). A conceptual model for inclusive technology: advancing disability inclusion through artificial intelligence. J. Disab. Res. 3:20230060. doi: 10.57197/JDR-2023-0060, 41948350

[ref2] Bae J. Lee S. Choi C. J. W. (2025). Expansion of digital technology use in the Korean social work field. J. Soc. Serv. Res. 51, 663–677. doi: 10.1080/01488376.2024.2402520

[ref3] Bandura A. (1997). Self-Efficacy: The Exercise of Control. New York, NY: W H Freeman/Times Books/ Henry Holt and Co.

[ref4] Belachew M. Adamu A. Ashete A. Mengistie S. (2025). The impact of digital empowerment training on the awareness of assistive technologies and digital competence skills among students with visual impairment. Disabil. Rehabil. Assist. Technol. 20, 2872–2884. doi: 10.1080/17483107.2025.2508395, 40397603

[ref5] Bentley S. V. Naughtin C. K. McGrath M. J. Irons J. L. Cooper P. S. (2024). The digital divide in action: how experiences of digital technology shape future relationships with artificial intelligence. AI Ethics 4, 901–915. doi: 10.1007/s43681-024-00452-3

[ref6] Berget G. MacFarlane A. (2020). What is known about the impact of impairments on information seeking and searching? J. Assoc. Inf. Sci. Technol. 71, 596–611. doi: 10.1002/asi.24256

[ref7] Cabero-Almenara J. Gutiérrez-Castillo J. J. Palacios-Rodríguez A. Guillén-Gámez F. D. (2022). Digital competence of university students with disabilities and factors that determine it. A descriptive, inferential and multivariate study. Educ. Inf. Technol. 28, 9417–9436. doi: 10.1007/s10639-022-11297-w

[ref8] Chemnad K. Othman A. (2024). Digital accessibility in the era of artificial intelligence—bibliometric analysis and systematic review. Front. Artif. Intell. 7:1349668. doi: 10.3389/frai.2024.1349668, 38435800 PMC10905618

[ref9] Cho M. Kim K. M. (2021). Exploring the disparity in tangible outcomes of internet use between persons with disabilities and persons without disabilities in South Korea. Disabil. Health J. 14:101101. doi: 10.1016/j.dhjo.2021.101101, 33824093

[ref10] Collins L. M. Lanza S. T. (2013). Latent Class and Latent Transition Analysis: With Applications in the Social, Behavioral, and Health Sciences. London: John Wiley and Sons.

[ref11] Dobransky K. Hargittai E. (2016). Unrealized potential: exploring the digital disability divide. Poetics 58, 18–28. doi: 10.1016/j.poetic.2016.08.003

[ref9001] Donat E. Brandtweiner R. Kerschbaum J. (2009). Attitudes and the Digital Divide: Attitude Measurement as Instrument to Predict Internet Usage. Informing Sci J. 12, 37–56. doi: 10.28945/427

[ref12] Getenet S. Cantle R. Redmond P. Albion P. (2024). Students’ digital technology attitude, literacy and self-efficacy and their effect on online learning engagement. Int. J. Educ. Technol. High. Educ. 21:3. doi: 10.1186/s41239-023-00437-y

[ref13] Giakoumis D. Kaklanis N. Votis K. Tzovaras D. (2014). Enabling user interface developers to experience accessibility limitations through visual, hearing, physical and cognitive impairment simulation. Univ. Access Inf. Soc. 13, 227–248. doi: 10.1007/s10209-013-0309-0

[ref14] Glumbić N. Đorđević M. Brojčin B. (2022). “Digital participation and disability digital divide,” in Digital Inclusion of Individuals with Autism Spectrum Disorder, eds. Glumbić N. Đorđević M. Brojčin B. (Cham: Springer International Publishing), 1–17.

[ref15] Heponiemi T. Gluschkoff K. Leemann L. Manderbacka K. Aalto A.-M. Hyppönen H. (2023). Digital inequality in Finland: access, skills and attitudes as social impact mediators. New Media Soc. 25, 2475–2491. doi: 10.1177/14614448211023007

[ref16] International Organization for Standardization (2018). ISO 9241-11:2018 ergonomics of human-system interaction—part 11: usability: definitions and concepts. Available online at: https://www.boutique.afnor.org/en-gb/standard/iso-9241112018/ergonomics-of-humansystem-interaction-part-11-usability-definitions-and-con/xs026028/125896 (accessed April 28, 2026).

[ref17] Khanlou N. Khan A. Vazquez L. M. Zangeneh M. (2021). Digital literacy, access to technology and inclusion for young adults with developmental disabilities. J. Dev. Phys. Disabil. 33, 1–25. doi: 10.1007/s10882-020-09738-w

[ref18] Kim M. Jung W. Kim S. Y. Park J. H. Shin D. W. (2023). The Korea National Disability Registration System. Epidemiol Health 45:e2023053. doi: 10.4178/epih.e2023053, 37189275 PMC10482564

[ref20] Lazic M. Simovic V. Domazet I. (2025). Digital competences and disability: a contribution to a more inclusive digital society. Univ. Access Inf. Soc. 24, 1623–1642. doi: 10.1007/s10209-024-01165-4

[ref21] Lubke G. Muthén B. O. (2007). Performance of factor mixture models as a function of model size, covariate effects, and class-specific parameters. Struct. Equ. Model. 14, 26–47. doi: 10.1080/10705510709336735

[ref22] Malik S. Elbatal I. Khan S. U. (2024). People with disabilities, the age of information and communication technology and the prevailing digital divide—a descriptive analysis. J. Disab. Res. 3:20240011. doi: 10.57197/JDR-2024-0011

[ref23] Ministry of Health and Welfare of Korea (2025). Current status of persons with disabilities. Available online at: https://kosis.kr/statHtml/statHtml.do?orgId=117andtblId=DT_11761_N008andconn_path=I2 (accessed March 13, 2026).

[ref24] Moon M. J. (2024). “Advancing Korea’s digital government: the shift from digital government to digital platform government,” in The Routledge International Handbook of Public Administration and Digital Governance, eds. Giest S. Roberge I. (New York, NY: Taylor and Francis), 62–78.

[ref25] Moreno L. Petrie H. Martínez P. Alarcon R. (2024). Designing user interfaces for content simplification aimed at people with cognitive impairments. Univ. Access Inf. Soc. 23, 99–117. doi: 10.1007/s10209-023-00986-z, 37361672 PMC10036960

[ref26] NIA (2025). The report on the digital divide (2024). Available online at: https://nia.or.kr/site/nia_kor/ex/bbs/View.do?cbIdx=81623andbcIdx=27832andparentSeq=27832 (accessed October 3, 2025).

[ref27] Nylund K. L. Asparouhov T. Muthén B. O. (2007). Deciding on the number of classes in latent class analysis and growth mixture modeling: a Monte Carlo simulation study. Struct. Equ. Model. 14, 535–569. doi: 10.1080/10705510701575396

[ref28] Pan X. (2020). Technology acceptance, technological self-efficacy, and attitude toward technology-based self-directed learning: learning motivation as a mediator. Front. Psychol. 11:564294. doi: 10.3389/fpsyg.2020.564294, 33192838 PMC7653185

[ref29] Park E.-Y. (2022). Digital competence and internet use/behavior of persons with disabilities in PC and smart device use. Univ. Access Inf. Soc. 21, 477–489. doi: 10.1007/s10209-020-00782-z

[ref30] Park E.-Y. (2025). Factors related to digital literacy in people with disabilities: focus on self-efficacy and attitude toward digital devices and technology. Int. Rev. Econ. Finance 104:104671. doi: 10.1016/j.iref.2025.104671

[ref31] Pettersson L. Johansson S. Demmelmaier I. Gustavsson C. (2023). Disability digital divide: survey of accessibility of eHealth services as perceived by people with and without impairment. BMC Public Health 23:181. doi: 10.1186/s12889-023-15094-z, 36707791 PMC9880913

[ref32] Rohde J. Marciniak M. A. Henninger M. Homan S. Ries A. Paersch C. . (2024). Effects of a digital self-efficacy training in stressed university students: a randomized controlled trial. PLoS One 19:e0305103. doi: 10.1371/journal.pone.0305103, 39480821 PMC11527301

[ref33] Sahib N. G. Tombros A. Stockman T. (2012). A comparative analysis of the information-seeking behavior of visually impaired and sighted searchers. J. Am. Soc. Inf. Sci. Tec. 63, 377–391. doi: 10.1002/asi.21696

[ref34] Spante M. Hashemi S. S. Lundin M. Algers A. (2018). Digital competence and digital literacy in higher education research: systematic review of concept use. Cogent Educ. 5:1519143. doi: 10.1080/2331186X.2018.1519143

[ref35] Tinmaz H. Lee Y.-T. Fanea-Ivanovici M. Baber H. (2022). A systematic review on digital literacy. Smart Learn. Environ. 9:21. doi: 10.1186/s40561-022-00204-y, 40478098 PMC9175160

[ref36] van Deursen A. Dijk J. (2011). Internet skills and the digital divide. New Media Soc. 13, 893–911. doi: 10.1177/1461444810386774

[ref37] Vereenooghe L. Trussat F. Baucke K. (2021). Applying the technology acceptance model to digital mental health interventions: a qualitative exploration with adults with intellectual disabilities. J. Ment. Health Res. Intellect. Disabil. 14, 318–343. doi: 10.1080/19315864.2021.1929597

[ref9002] Vicente M. R. López A. J. (2010). A Multidimensional Analysis of the Disability Digital Divide: Some Evidence for Internet Use. Inform. Soc. 26, 48–64. doi: 10.1080/01615440903423245

[ref38] Vuorikari R. Kluzer S. Punie Y. (2022). DigComp 2.2, The Digital Competence Framework for Citizens: with new Examples of Knowledge, Skills and Attitudes. Lubowa: LU Publications Office.

[ref39] Yoon H. J. Kim Y. S. (2025). Latent profiles and predictors of transfer-assistive robot acceptance among Korean care workers. Innov. Aging 9:igaf122.4306. doi: 10.1093/geroni/igaf122.4306

[ref40] Yu T.-Y. Liu C.-H. Horng J.-S. Chou S.-F. Huang Y.-C. Fang Y.-P. . (2025). Discovering how digital attitudes, control, self-efficacy and social norms influence the digital behavior decision-making of leisure and recreation activities participants. Curr. Psychol. 44, 1032–1054. doi: 10.1007/s12144-024-07163-2

[ref41] Zhou K. Kim J. Choi H. (2025). A person-centred, latent profile analysis of assistive technology needs among autistic adults in employment. Disabil. Rehabil. Assist. Technol. 1–16. doi: 10.1080/17483107.2025.2583314, 41208536

