# Supplementary material for: Digital application competence among adults with disabilities in South Korea: associations with latent profiles of digital attitudes and digital self-efficacy
Source: Front Psychol. 2026 Jun 10;17:1830991. doi: 10.3389/fpsyg.2026.1830991 (PMC13291150; doi:10.3389/fpsyg.2026.1830991)
Supplement: Supplementary file 1 [file Table_1.DOCX]

Supplementary Material

# Table S1. Average posterior probability.

|  | Low Attitudes–Low Self-Efficacy | Moderate Attitudes–Moderate Self-Efficacy | High Attitudes–Low Self-Efficacy | High Attitudes–High Self-Efficacy | Very High Attitudes-Very High Self-Efficacy |
| --- | --- | --- | --- | --- | --- |
| Low Attitudes–Low Self-Efficacy | 0.963 | 0.024 | 0.013 | 0.000 | 0.000 |
| Moderate Attitudes–Moderate Self-Efficacy | 0.015 | 0.909 | 0.040 | 0.036 | 0.000 |
| High Attitudes–Low Self-Efficacy | 0.007 | 0.056 | 0.910 | 0.026 | 0.000 |
| High Attitudes–High Self-Efficacy | 0.000 | 0.017 | 0.028 | 0.944 | 0.012 |
| Very High Attitudes–Very High Self-Efficacy | 0.000 | 0.000 | 0.000 | 0.090 | 0.910 |

# Table S2. Multinomial logistic regression of latent profile membership of digital attitudes and digital self-efficacy.

| Variables | Model 1 | Model 2 | Model 3 | Model 4 |
| --- | --- | --- | --- | --- |
|  | Low Attitudes–Low Self-Efficacy | Moderate Attitudes–Moderate Self-Efficacy | High Attitudes–Low Self-Efficacy | Very High Attitudes–Very High Self-Efficacy |
|  | (ref. High Attitudes–High Self-Efficacy) | | | |
| Age | 1.05*** (1.03 - 1.07) | 1.05*** (1.04 -1.06) | 1.05***(1.03 - 1.06) | 0.97*** (0.95 - 0.98) |
| Male (ref. Female) | 0.74 (0.51 - 1.08) | 1.04 (0.78 - 1.37) | 0.78* (0.60 - 1.01) | 1.20 (0.83 - 1.75) |
| Education (ref. Primary school or below) | | | | |
| Middle school | 0.29*** (0.11 - 0.74) | 0.45* (0.18 - 1.13) | 0.39** (0.16 - 0.95) | 0.23** (0.06 - 0.97) |
| High school | 0.09*** (0.03 - 0.22) | 0.28*** (0.11 - 0.68) | 0.22*** (0.10 - 0.51) | 0.26** (0.07 - 0.92) |
| University or above | 0.003*** (0.00 - 0.03) | 0.10*** (0.04 - 0.26) | 0.08*** (0.03 - 0.20) | 0.57 (0.16 - 2.07) |
| Urban (ref. Rural) | 1.15 (0.71 - 1.84) | 2.43*** (1.57 - 3.77) | 1.10 (0.79 - 1.54) | 1.04 (0.64 - 1.68) |
| Income | 0.76*** (0.67 - 0.86) | 0.95 (0.88 - 1.03) | 0.83*** (0.77 - 0.90) | 0.95 (0.86 - 1.04) |
| Disability type (Ref. PI) | | | | |
| VI | 1.36 (0.72 - 2.56) | 1.58** (1.08 - 2.31) | 1.39* (0.96 - 2.00) | 1.13 (0.73 - 1.75) |
| BI | 2.84*** (1.69 - 4.76) | 1.81*** (1.21 - 2.70) | 1.99*** (1.37 - 2.90) | 0.62 (0.34 - 1.14) |
| HSI | 1.49 (0.90 - 2.47) | 1.11 (0.74 - 1.68) | 1.08 (0.73 - 1.59) | 1.37 (0.83 - 2.25) |
| Congenital (ref. Acquired) | 1.49** (1.01 - 2.20) | 1.23 (0.92 - 1.65) | 0.98 (0.73 - 1.30) | 0.49*** (0.32 - 0.75) |
| Severe (ref. Mid) | 1.65*** (1.13 - 2.41) | 1.80*** (1.37 - 2.36) | 1.68*** (1.29 - 2.18) | 2.05*** (1.44 - 2.91 |

^*^*p* < 0.05, ^**^*p* < 0.01, ^***^*p* < 0.001; PI = Physical Impairment; VI = Visual Impairment; BI = Brain Impairment; HSI = Hearing/speech Impairment; Values outside parentheses are odds ratios (ORs), and values inside parentheses are 95% confidence intervals (CIs).
